# Body mass index, mammographic density, and breast cancer risk by estrogen receptor subtype

**DOI:** 10.1186/s13058-019-1129-9

**Published:** 2019-04-03

**Authors:** Yiwey Shieh, Christopher G. Scott, Matthew R. Jensen, Aaron D. Norman, Kimberly A. Bertrand, V. Shane Pankratz, Kathleen R. Brandt, Daniel W. Visscher, John A. Shepherd, Rulla M. Tamimi, Celine M. Vachon, Karla Kerlikowske

**Affiliations:** 1 Division of General Internal Medicine, University of California, San Francisco, 1545 Divisadero Street, Box 0320, San Francisco, CA 94115 USA; 2 Department of Health Sciences Research, Mayo Clinic, Rochester, MN USA; 3 Slone Epidemiology Center at Boston University, Boston, MA USA; 4 Department of Internal Medicine, University of New Mexico, Albuquerque, NM USA; 5 Department of Radiology, Mayo Clinic, Rochester, MN USA; 6 Department of Laboratory Medicine and Pathology, Mayo Clinic, Rochester, MN USA; 7 Department of Radiology, University of Hawaii Cancer Center, Honolulu, HI USA; 8 Channing Division of Network Medicine, Brigham and Women’s Hospital & Department of Epidemiology, Harvard T.H. Chan School of Public Health, Boston, MA USA; 9 General Internal Medicine Section, San Francisco Veterans Affairs Medical Center & Departments of Medicine and Epidemiology and Biostatistics, University of California, San Francisco, San Francisco, CA USA

**Keywords:** Breast neoplasms, Epidemiology, Risk factors, Mammographic breast density, Prevention

## Abstract

**Background:**

Obesity and elevated breast density are common risk factors for breast cancer, and their effects may vary by estrogen receptor (ER) subtype. However, their joint effects on ER subtype-specific risk are unknown. Understanding this relationship could enhance risk stratification for screening and prevention. Thus, we assessed the association between breast density and ER subtype according to body mass index (BMI) and menopausal status.

**Methods:**

We conducted a case-control study nested within two mammography screening cohorts, the Mayo Mammography Health Study and the San Francisco Bay Area Breast Cancer SPORE/San Francisco Mammography Registry. Our pooled analysis contained 1538 ER-positive and 285 ER-negative invasive breast cancer cases and 4720 controls matched on age, menopausal status at time of mammogram, and year of mammogram. Percent density was measured on digitized film mammograms using computer-assisted techniques. We used polytomous logistic regression to evaluate the association between percent density and ER subtype by BMI subgroup (normal/underweight, < 25 kg/m^2^ versus overweight/obese, ≥ 25 kg/m^2^). We used Wald chi-squared tests to assess for interactions between percent density and BMI. Our analysis was stratified by menopausal status and hormone therapy usage at the time of index mammogram.

**Results:**

Percent density was associated with increased risk of overall breast cancer regardless of menopausal status or BMI. However, when analyzing breast cancer across ER subtype, we found a statistically significant (*p* = 0.008) interaction between percent density and BMI in premenopausal women only. Specifically, elevated percent density was associated with a higher risk of ER-negative than ER-positive cancer in overweight/obese premenopausal women [OR per standard deviation increment 2.17 (95% CI 1.50–3.16) vs 1.33 (95% CI 1.11–1.61) respectively, *P*_heterogeneity_ = 0.01]. In postmenopausal women, elevated percent density was associated with similar risk of ER-positive and ER-negative cancers, and no substantive differences were seen after accounting for BMI or hormone therapy usage.

**Conclusions:**

The combination of overweight/obesity and elevated breast density in premenopausal women is associated with a higher risk of ER-negative compared with ER-positive cancer. Eighteen percent of premenopausal women in the USA have elevated BMI and breast density and may benefit from lifestyle modifications involving weight loss and exercise.

**Electronic supplementary material:**

The online version of this article (10.1186/s13058-019-1129-9) contains supplementary material, which is available to authorized users.

## Introduction

Breast cancer risk is multifactorial, with > 40% of women having multiple risk factors [1]. Moreover, breast cancer is biologically heterogeneous, with the main distinction being estrogen receptor (ER) status. Risk prediction models can be used to guide decisions around screening and prevention [2–5], but do not currently account for interactions between most risk factors or estimate ER subtype-specific risk.

Elevated breast density and overweight/obesity are the two most prevalent risk factors for breast cancer [1]. Breast density represents the relative amounts of dense (fibroglandular) and non-dense (fatty) areas on a mammogram. Of density measures, percent density, or the ratio of dense to overall breast area, is most strongly associated with risk [6]. Percent density and BMI are inversely related and act as confounders of each other’s effects [6, 7]. Breast density [8–10] and BMI [10] have been differentially associated with ER-positive and ER-negative cancers, and these associations vary by menopausal status [10]. For instance, premenopausal elevated percent breast density is associated with higher subsequent risk of ER-negative than ER-positive cancer, whereas postmenopausal elevated percent density is associated with similar risk of both ER subtypes [8]. Similarly, premenopausal overweight/obesity is associated with higher risk of ER-negative than ER-positive cancers, whereas postmenopausal overweight/obesity is associated with similar risk of both ER subtypes [10, 11]. The latter relationships are modified by postmenopausal hormone therapy (HT) use [10, 11].

An important clinical question is whether elevated breast density and overweight/obesity act to synergistically increase risk. Whereas recent work has estimated the population-wide effects of breast density and BMI [1], few studies have investigated how these risk factors interact. Three of four studies that tested for interactions between breast density and BMI found a synergistic interaction [12–14], with two being of borderline statistical significance [13, 14]. The remaining study found no interaction [15]. All studies shared two limitations: they included relatively few premenopausal women and did not stratify their results by ER status [12–15].

We hypothesized that the combined effects of elevated breast density and overweight/obesity exceed their individual effects. We further hypothesized that these differences would vary by menopausal status and ER subtype. Therefore, we examined the individual and combined effects of percent breast density and overweight/obesity on ER subtype-specific risk within two screening cohorts.

## Methods

### Study design and populations

We conducted a nested case-control study within two pooled cohorts, the Mayo Mammography Health Study (MMHS) [16, 17] and the San Francisco Bay Area Breast Cancer SPORE/San Francisco Mammography Registry (SFMR) [18, 19], Additional file 1: Figure S1. Eligible women undergoing routine screening mammography were recruited between 1996–2007 (SFMR) and 2003–2006 (MMHS).

Cases were women diagnosed with invasive breast cancer, as ascertained by linkage to the California Cancer Registry (SFMR), clinic- or state-based cancer registries (MMHS), or from pathology reports (MMHS). ER status was ascertained by immunohistochemical staining. To limit prevalent cases, we included breast cancers diagnosed > 6 months after the index mammogram used for density measurement. We excluded 590 cases due to missing data; of these, 50 were missing ER status (Additional file 1: Figure S1).

Controls were women from the underlying cohorts who were free of breast cancer at the time of most recent case ascertainment. Controls and cases were matched on age and menopausal status at the time of index mammogram, as well as the year of the screening examination. Matching also accounted for state of residence (MMHS) and screening facility (SFMR). The study was approved by the institutional review boards of the University of California, San Francisco, and the Mayo Clinic. Participants provided passive permission (SFMR) or informed consent (MMHS) for use of data.

### Mammographic breast density

Breast density measurements were performed on digitized images of pre-diagnostic film screening mammograms. We measured percent density using Cumulus, a computer-assisted threshold technique [20], and USCF custom mammographic density software [21]. Both techniques, which are highly correlated [21], use a threshold to define the dense and non-dense regions on the image. Percent density is the ratio of dense to total area. Measurements were performed on the craniocaudal view of the contralateral breast for cases and on the corresponding side for matched controls. We standardized density measures to remove variability related to age distributions of different study populations, readers, and times of density assessment [8].

### Other covariate measures

Menopausal status and postmenopausal HT use were ascertained through questionnaire responses collected at the time of index mammogram. Postmenopausal women were those who had both ovaries removed, whose periods had stopped naturally, were currently using HT, or were age 55 or older. Premenopausal women reported a period < 180 days ago, were under age 40, or were currently using birth control. Due to differing associations between combined hormonal and unopposed estrogen on breast cancer risk [22], HT users were defined as those actively taking estrogen alone or estrogen plus progestin at the time of the mammogram. We ascertained BMI (kg/m^2^) at the time of index mammogram using questionnaire response or medical record review.

### Statistical analysis

We constructed multivariable logistic regression models to evaluate the individual and combined effects of BMI and percent density on invasive breast cancer (overall and by ER subtype). To evaluate ER subtypes, polytomous (multinomial) logistic regression models were fit with three outcomes: no cancer (reference), ER-positive cancer, and ER-negative cancer. Our main predictor was percent density. We present the odds ratio (OR) and corresponding 95% confidence interval (CI) per standard deviation (SD) increment of standardized percent density, after square root transformation.

In overall analyses of ER-positive and ER-negative cancer, we tested for evidence of interactions among BMI categories, menopausal status (premenopausal, postmenopausal HT use, and postmenopausal HT non-use), and percent density by fitting appropriate multiplicative interaction terms. Based on the interaction test results, we performed stratified analyses according to menopausal status and BMI. For our main analysis, we dichotomized BMI into normal/underweight (< 25) and overweight/obese (≥ 25) subgroups due to sample size constraints. In supplemental analyses, we divided BMI into normal/underweight (< 25), overweight (25–29.9), and obese (≥ 30) subgroups to assess trends with increasing BMI.

All models were adjusted for age and study. Individual associations between BMI and breast cancer (overall and by ER subtype) were performed with and without adjustment for percent density. To account for residual confounding from differences in BMI within BMI subgroups, we performed supplemental analysis adjusting for continuous BMI within each BMI subgroup. Associations between percent density and breast cancer (overall and by ER subtype) were stratified by BMI subgroup, with additional adjustment for continuous BMI within BMI subgroups.

Contrasts were constructed within the polytomous logistic model framework to test for heterogeneity of associations between density measures and ER subtypes within BMI subgroups (*P*_het_). Similar contrasts were used to test for heterogeneity of association of BMI subgroups by ER subtypes. We used Wald chi-squared tests to examine multiplicative interaction effects between BMI subgroups and continuous percent density (*P*_int_) on subtype-specific risk within each menopausal stratum. Differences in associations between breast density and breast cancer for the BMI subgroups were evaluated by estimating an ordinal density trend within each subgroup and then testing for differences in these trends between BMI subgroups as a multiplicative interaction. To examine for confounding by race and time since prior mammogram, additional analyses were performed in Caucasians only and with additional adjustment for year of index mammogram. All tests were two-sided with *α* = 0.05. We performed the analyses using SAS software (version 9.4, SAS Institute).

### Estimation of risk factor prevalence

We estimated the prevalence of overweight/obesity and elevated breast density in the US population using data from the Breast Cancer Surveillance Consortium (BCSC) Risk Factors Dataset [23]. This database includes detailed risk factor information for 1,144,564 women undergoing mammography screening at BCSC imaging centers between 2000 and 2009. Breast density was reported according to Breast Imaging Reporting and Data System (BI-RADS) categories: a = almost entirely fatty; b = scattered fibroglandular densities; c = heterogeneously dense, and d = extremely dense [24]. We considered BI-RADS categories c and d to represent elevated breast density since they correspond to area-based percent density > 50% [25], approximately one standard deviation above the mean percent density in controls within our study.

## Results

We included 1538 ER-positive and 285 ER-negative breast cancer cases and 4720 controls (Table 1 and Additional file 1: Table S1). The median time from index mammogram to diagnosis for cases was 4.2 years (interquartile range 2.8–5.5 years). Cases had higher percent density and BMI than controls. Nearly 1/3 of women were premenopausal at the time of index mammogram.

**Table 1:** Baseline characteristics of 6543 women included in nested case-control analysis

Characteristic	Cases^a^ (*n* = 1823)	Controls^a^ (*n* = 4720)
Age at diagnosis, years (median, IQR)	60 (52–69)	
Age at mammogram, years (median, IQR)	56 (48–65)	55 (48–65)
Race/ethnicity, No. (%)		
Caucasian	1330 (73.0)	3365 (71.3)
Asian	301 (16.5)	844 (17.9)
Hispanic/Latina	69 (3.8)	143 (3.0)
African-American	69 (3.8)	199 (4.2)
Multiracial	34 (1.8)	121 (2.6)
Other (not falling in categories above)	20 (1.1)	48 (1.0)
BMI, kg/m^2^ (median, IQR)	24.8 (22.0–28.9)	24.2 (21.6–27.6)
Menopausal status^a^		
Premenopausal	610 (33.5%)	1507 (31.9%)
Postmenopausal, estrogen use	140 (7.7%)	435 (9.2%)
Postmenopausal, estrogen + progestin use	339 (18.6%)	689 (14.6%)
Postmenopausal, no HT use	734 (40.3%)	2089 (44.3%)
Positive family history of breast cancer, No. (%)	404 (22.2%)	712 (15.1%)
Percent density (median, IQR)	31.8 (16.9–48.4)	24.7 (12.0–41.0)

Abbreviations: *BMI* body mass index, *HT* hormone therapy, *IQR* interquartile range (25th, 75th percentile)

^a^Controls and cases were matched on age, menopausal status at the time of index mammogram, and year of examination

Interaction tests revealed evidence of differential ER subtype associations for BMI by menopausal categories (*p* = 0.02) and percent density by BMI categories (*p* = 0.04). Based on these interactions, we evaluated the associations between overweight/obese BMI and breast cancer by ER subtype, across menopausal status (Table 2). After adjustment for percent density, premenopausal women who were overweight/obese had increased risk of ER-negative cancer [OR 2.51 (95% CI 1.61–3.93)] and ER-positive cancer [OR 1.53 (95% CI 1.21–1.94)] relative to their normal/underweight counterparts. The heterogeneity of ER subtype-specific risk for overweight/obese women reached statistical significance (*P*_het_ = 0.04). Postmenopausal HT non-users who were overweight/obese also had increased risk of ER-negative cancer [OR 1.62 (95% CI 1.06–2.94)] and ER-positive cancer [OR 2.28 (95% CI 1.86–2.81)] relative to their normal/underweight counterparts, though the heterogeneity of ER subtype-specific risk did not reach statistical significance (*P*_het_ = 0.13). Postmenopausal HT users who were overweight or obese had increased risk of ER-positive cancers only [OR 1.58 (95% CI 1.23–2.04)].

**Table 2:** Associations of overweight/obese BMI with ER-positive and ER-negative breast cancer, by menopausal status for 1823 cases and 4720 controls

	Normal/underweight BMI < 25 kg/m^2^			Overweight/obese BMI ≥ 25 kg/m^2^					
	Cases	Controls	OR (95% CI)	Cases	Controls	BMI association (unadjusted for density) OR (95% CI)^a^	*P*_het_^c^	BMI association (adjusted for density) OR (95% CI)^b^	*P*_het_^c^
Premenopausal							0.07		0.04
ER-positive cancer	325	979	1.00 (ref)	180	528	1.06 (0.86–1.32)		1.53 (1.21–1.94)	
ER-negative cancer	58	979	1.00 (ref)	47	528	1.58 (1.05–2.38)		2.51 (1.61–3.93)	
Postmenopausal HT user							0.95		0.75
ER-positive cancer	240	678	1.00 (ref)	171	446	1.16 (0.92–1.47)		1.58 (1.23–2.04)	
ER-negative cancer	41	678	1.00 (ref)	27	446	1.14 (0.69–1.90)		1.44 (0.84–2.48)	
Postmenopausal HT non-user							0.24		0.13
ER-positive cancer	228	1029	1.00 (ref)	394	1060	1.69 (1.40–2.04)		2.28 (1.86–2.81)	
ER-negative cancer	51	1029	1.00 (ref)	61	1060	1.32 (0.89–1.94)		1.62 (1.06–2.47)	

Abbreviations: *BMI* body mass index, *CI* confidence interval, *ER* estrogen receptor, *HT* hormone therapy, *OR* odds ratio

^a^Odds ratios estimated from polytomous multivariable logistic regression models adjusted for age, study

^b^Odds ratios estimated from polytomous multivariable logistic regression models adjusted for age, study, and percent density

^c^*P* value of heterogeneity of BMI by subtype association

Next, we evaluated the associations between percent density and breast cancer by ER subtype (Additional file 1: Table S2). Elevated percent density was associated with increased risk of both ER-positive and ER-negative cancers in premenopausal and postmenopausal women, although the association between percent density and ER-negative cancers was of borderline significance in HT users.

We then evaluated the associations between percent density and overall invasive breast cancer across BMI and menopausal status subgroups (Table 3). Elevated percent density was associated with increased risk across BMI subgroups. In premenopausal women, the effect of BMI-adjusted density was somewhat stronger in normal/underweight women compared to overweight/obese women [OR 1.83 (95% CI 1.54–2.18) vs 1.46 (95% CI 1.22–1.74)]. The test for interaction between BMI and percent density was of borderline statistical significance (*P*_int_ = 0.07). In postmenopausal HT non-users and users, the associations between percent density and overall breast cancer were similar across BMI subgroups. Adjusting for residual confounding by BMI within BMI subgroups did not substantively change the results (data not shown).

**Table 3:** Associations of percent density with overall breast cancer, by BMI and menopausal status for 1823 cases and 4720 controls

	Normal/underweight BMI < 25 kg/m^2^			Overweight/obese BMI ≥ 25 kg/m^2^			
	Cases	Controls	Percent density OR per S.D. (95% CI)^a^	Cases	Controls	Percent density OR per S.D. (95% CI)^a^	*P*_int_^b^
Premenopausal	383	979	1.83 (1.54–2.18)	227	528	1.46 (1.22–1.74)	0.07
Postmenopausal HT user	281	678	1.64 (1.38–1.95)	198	446	1.61 (1.32–1.95)	0.84
Postmenopausal HT non-user	279	1029	1.46 (1.24–1.70)	455	1060	1.48 (1.30–1.69)	0.94

Abbreviations: *BMI* body mass index, *CI* confidence interval, *ER* estrogen receptor, *HT* hormone therapy, *OR* odds ratio, *S.D.* standard deviation

^a^Odds ratios estimated from polytomous multivariable logistic regression models adjusted for age, study. Standard deviation of square root-transformed percent density = 2.0

^b^*P* value of test for BMI-percent density interaction

When we analyzed breast cancer by ER subtype, we observed a statistically significant, synergistic interaction between overweight/obesity and elevated percent density on ER-specific risk in premenopausal women (*P*_int_ = 0.008), Table 4. Specifically, in premenopausal overweight/obese women, elevated percent density was associated with higher risk of ER-negative compared to ER-positive cancer [OR 2.17 (95% CI 1.50–3.16) vs. 1.33 (95% CI 1.11–1.61) respectively, *P*_het_ = 0.01]. In contrast, among normal/underweight women, elevated percent density tended to be associated with *lower* risk of ER-negative compared to ER-positive cancer [OR per standard deviation increment 1.51 (95% CI 1.04–2.21) vs 1.90 (95% CI 1.57–2.29), *P*_het_ = 0.27]. The interaction between percent density and BMI remained after adjusting for residual confounding within strata (data not shown), and when analyzing BMI across normal/underweight, overweight, and obese subgroups (Additional file 1: Table S3).

**Table 4:** Associations of percent density with ER-positive and ER-negative breast cancer, by BMI and menopausal status for 1823 cases and 4720 controls

	Normal/underweight BMI < 25 kg/m^2^				Overweight/obese BMI ≥ 25 kg/m^2^			
	Cases	Controls	Percent density OR per S.D. (95% CI)^a^	*P*_het_^b^	Cases	Controls	Percent density OR per S.D. (95% CI)^a^	*P*_het_^b^
Premenopausal^c^				0.27				0.01
ER-positive cancer	325	979	1.90 (1.57–2.29)		180	528	1.33 (1.11–1.61)	
ER-negative cancer	58	979	1.51 (1.04–2.21)		47	528	2.17 (1.50–3.16)	
Postmenopausal HT user^d^				0.22				0.89
ER-positive cancer	240	678	1.71 (1.42–2.05)		171	446	1.60 (1.30–1.96)	
ER-negative cancer	41	678	1.34 (0.92–1.94)		27	446	1.66 (1.06–2.60)	
Postmenopausal HT non-user^e^				0.25				0.66
ER-positive cancer	228	1029	1.51 (1.27–1.80)		394	1060	1.50 (1.31–1.71)	
ER-negative cancer	51	1029	1.23 (0.89–1.70)		61	1060	1.40 (1.04–1.87)	

Abbreviations: *BMI* body mass index, *CI* confidence interval, *ER* estrogen receptor, *HT* hormone therapy, *OR* odds ratio, *S.D.* standard deviation

^a^Odds ratios estimated from polytomous multivariable logistic regression models adjusted for age, study. Standard deviation of square root-transformed percent density = 2.0

^b^*P* value of heterogeneity of association between percent density and ER subtypes within BMI groups

^c^*P*_int_ = 0.008 for BMI-percent density interaction within premenopausal women

^d^*P*_int_ = 0.58 for BMI-percent density interaction within postmenopausal HT users

^e^*P*_int_ = 0.89 for BMI-percent density interaction within postmenopausal HT non-users

We detected no interactions between percent density and BMI in postmenopausal women (Table 4). Associations between percent density and ER-positive risk were statistically significant across BMI subgroups in both HT users and non-users. The associations were generally comparable to those seen for ER-negative cancers, though the latter associations only reached statistical significance among overweight/obese HT users and non-users. Similarly, no evidence of interaction was seen after adjusting for BMI as a continuous variable (data not shown) or analyzing BMI across normal/underweight, overweight, and obese subgroups (Additional file 1: Table S3).

The interaction between overweight/obesity and percent density across ER subtype-specific risk was also observed in premenopausal women when the analysis was restricted to Caucasian women (Additional file 1: Table S4). Adjusting for year of index mammogram also did not change the results (Additional file 1: Table S5).

We estimated the prevalence of elevated breast density and overweight/obesity in the US population using a population-based database of over 1.1 million women undergoing mammography screening in the USA [23]. Forty-five percent of premenopausal women had dense breasts (defined as BI-RADS c or d), 47% were overweight/obese, and 18% had both risk factors.

## Discussion

We investigated the individual and combined effects of two prevalent risk factors, overweight/obesity and percent density, on ER subtype-specific risk. We specifically examined whether these two risk factors interact, and whether interactions differ based on menopausal status. We found that premenopausal overweight/obesity primarily increases the risk of ER-negative cancer, whereas postmenopausal overweight/obesity increases the risk of ER-positive cancer in HT non-users. Notably, the effects of elevated percent density on ER subtype-specific risk vary by BMI. Specifically, elevated percent density interacts synergistically with overweight/obesity to increase ER-negative cancer risk in premenopausal women. We estimate that approximately 18% of premenopausal women are overweight/obese *and* have elevated breast density.

The main implication of our findings is that BMI acts an effect modifier for percent density in premenopausal women, with the effect varying by ER subtype. Our findings expand the literature, which had previously evaluated the BMI-percent density interaction in the context of overall breast cancer risk only. A prior analysis of 1699 cases and 2422 controls reported a BMI-density interaction of borderline statistical significance (*P*_int_ = 0.06) for overall breast cancer. There was no evidence of interaction in premenopausal women (*P*_int_ = 0.32), although this group comprised < 25% of the study [14]. Our study included more premenopausal women and analyzed breast cancer by ER status, which may have enhanced our ability to detect interactions.

In contrast, we found no interactions between percent density and BMI in postmenopausal women. This result is consistent with an analysis of postmenopausal women from the Nurses’ Health Study, where BMI did not modify the association between percent density and overall breast cancer (*P*_int_ = 0.92) [15]. Though a synergistic interaction between percent density categories and BMI was seen in the Singapore Breast Screening Project, the interaction was not statistically significant when percent density was analyzed as a continuous variable [13].

Our findings contribute to the evolving understanding of BMI’s effect on ER-negative cancer risk. We replicate findings from case-control [10] and case-case [26] studies showing a positive association between premenopausal overweight/obesity and ER-negative cancer. Similarly, overweight BMI was associated with ER-negative cancers within premenopausal participants in the National Surgical Adjuvant Breast and Bowel Project P-1 Trial chemoprevention trial [HR 2.52 (95% 1.19–5.33)]. No association was seen in obese women, though there were few ER-negative cases [27]. Beyond ER-negative cancers, obesity has also been associated with premenopausal triple-negative cancers [28, 29]. Taken together, there is growing evidence that premenopausal overweight/obesity may increase the risk of ER-negative and triple-negative cancers. The underlying mechanisms are poorly understood, but may involve chronic inflammation or insulin resistance [28, 30]. Whereas most prior studies found premenopausal overweight/obesity to be inversely associated with breast cancer, particularly ER-positive cancers, these studies primarily analyzed premenopausal cancers. Since premenopausal cancers arise more proximally to BMI ascertainment compared with postmenopausal cancers, the timing of BMI ascertainment relative to cancer diagnosis may explain discrepancies in the literature.

One unexpected finding was that premenopausal normal/underweight women, compared with their overweight/obese counterparts, had substantially elevated ER-positive risk commensurate with increased breast density. Whereas overweight/obesity is thought to increase ER-positive cancer through peripheral aromatization of estrogens by adipose tissue and through insulin signaling [30], our finding of increased ER-positive risk in normal/underweight women with dense breasts merits further investigation.

Our study has several notable strengths. Since we assessed BMI at the time of pre-diagnostic mammogram, rather than at the time of diagnosis, we could assess the downstream risk associated with being overweight/obese. Our results describe the distal effects of premenopausal overweight/obesity, which is of greater relevance for risk stratification than overweight/obesity immediately prior to diagnosis. Other strengths include the ability to leverage two established mammography cohorts containing quantitative, area-based density measurements and detailed tumor characteristics assessed from cancer registries or institutional records. Although participant characteristics differed somewhat between cohorts, we have previously shown that breast density, our main predictor, displays similar associations with breast cancer across MMHS and SFMR [8].

Our study has several limitations. First, although our BMI measurements preceded diagnosis by a median of 5 years, we had limited ability to measure longitudinal changes in BMI before and after the index mammogram. The duration of exposure to increased BMI, and trajectory of BMI, may both affect risk. For instance, adult weight gain has been found to modify the associations between BMI and ER subtype-specific risk in some studies [31, 32], but we did not ascertain early adulthood BMI. Second, the number of ER-negative cancers in our study limited the precision of our estimated associations. Third, since we tested for interactions between BMI and density across menopausal strata, some of our associations could be due to type I error. However, we sought to mitigate multiple comparisons and increase power by analyzing density as a continuous, rather than categorical, variable. Fourth, we measured breast density using Cumulus, an area-based measurement, whereas available commercial measures such as Volpara and Quantra use volumetric measures that are not modified by BMI [33]. Thus, our results should be replicated with automated volumetric measures. Lastly, our findings may not be generalizable to the US population at large given that the median BMI in our study population was 24.3, compared to 28.0–28.5 for US women aged 40–69 [34], and our study contained relatively few African-American and Latina women.

If our findings are replicated across other volumetric density measures, and in diverse populations, they may have important implications for risk stratification in premenopausal women, 18% of whom have BI-RADS category heterogeneously dense or extremely dense breast density and BMI ≥ 25, according to our analysis of a large database of US women undergoing mammography screening. These women may benefit from counseling on their risk for ER-negative cancers and the importance of achieving and maintaining a normal BMI. Most studies on the relationship between weight trajectory and breast cancer risk have focused on postmenopausal women, with several showing that weight loss decreases risk [35–37] and others failing to detect an association [38]. Little is known about premenopausal weight and breast cancer risk, or how weight trajectory and lifestyle factors such as exercise impact subtype-specific risk. Since selective estrogen receptor modulators such as tamoxifen and raloxifene do not reduce the risk of ER-negative cancers [39], lifestyle modification could be a critical strategy to reduce ER-negative cancer risk in young women with dense breasts. For instance, increased physical activity has been consistently linked to decreased breast cancer incidence [RR 0.87 (95% CI 0.84–0.90) in a recent umbrella review] [40], and it is highly plausible that such an effect is mediated by a reduction in BMI [41].

Our findings could potentially be leveraged to enhance decision-making around chemoprevention, given the observed association between percent density and ER-positive cancers in normal/underweight women. The BCSC [4, 42] and Tyrer-Cuzick [43] risk models include breast density, and efforts to modify them to provide ER subtype-specific risk should consider BMI-density interactions. Given the notable differences in breast cancer subtypes by race/ethnicity [44], for instance, the elevated incidence of ER-negative cancers in African-Americans [45], future work should assess whether African-American and Latina women exhibit similar associations between density, BMI, and subtype-specific risk.

## Conclusions

Elevated breast density and premenopausal overweight/obesity are risk factors for ER-negative breast cancer and interact synergistically to augment ER-negative risk. Both risk factors coexist in a substantial proportion of premenopausal women. Our findings demonstrate how prevalent, commonly captured risk factors can enhance understanding of overall and phenotypic breast cancer risk. Premenopausal women at elevated risk of ER-negative cancer represent a population where further studies on weight loss and physical activity interventions are especially needed.

## Additional file

Additional file 1: **Figure S1.** Flow diagram of subjects included in pooled analysis. **Table S1.** Baseline characteristics of 6543 women, by study. **Table S2.** Associations of percent density with ER-positive and ER-negative invasive breast cancer, by menopausal status for 1823 cases and 4720 controls. **Table S3.** Associations of percent density with ER-positive and ER-negative breast cancer, by BMI (normal/underweight, overweight, obese) and menopausal status for 1823 cases and 4720 controls. **Table S4.** Associations of percent density with ER-positive and ER-negative breast cancer, by BMI and menopausal status for 1330 Caucasian cases and 3365 Caucasian controls. **Table S5.** Associations of percent density with ER-positive and ER-negative breast cancer, by BMI and menopausal status, and adjusted for year of index mammogram, for 1823 cases and 4720 controls. (DOCX 2426 kb)

## Abbreviations

BCSC: Breast Cancer Surveillance Consortium
BI-RADS: Breast Imaging Reporting and Data System
BMI: Body mass index
CI: Confidence interval
ER: Estrogen receptor
HT: Hormone therapy
MMHS: Mayo Mammography Health Study
OR: Odds ratio
SD: Standard deviation
SFMR: San Francisco Mammography Registry
