# Supplementary material for: Body mass index, mammographic density, and breast cancer risk by estrogen receptor subtype
Source: Breast Cancer Res. 2019 Apr 3;21:48. doi: 10.1186/s13058-019-1129-9 (PMC6448282; doi:10.1186/s13058-019-1129-9)
Supplement: Supplementary file 1 — Figure S1. Flow diagram of subjects included in pooled analysis. Table S1. Baseline characteristics of 6543 women, by study. Table S2. Associations of percent density with ER-positive and ER-negative invasive breast cancer, by menopausal status for 1823 cases and 4720 controls. Table S3. Associations of percent density with ER-positive and ER-negative breast cancer, by BMI (normal/underweight, overweight, obese) and menopausal status for 1823 cases and 4720 controls. Table S4. Associations of percent density with ER-positive and ER-negative breast cancer, by BMI and menopausal status for 1330 Caucasian cases and 3365 Caucasian controls. Table S5. Associations of percent density with ER-positive and ER-negative breast cancer, by BMI and menopausal status, and adjusted for year of index mammogram, for 1823 cases and 4720 controls. (DOCX 2426 kb) [file 13058_2019_1129_MOESM1_ESM.docx]

**Additional file 1**

**Body mass index, mammographic density, and breast cancer risk by estrogen receptor subtype**

**Authors:**

Yiwey Shieh, MD

Christopher G. Scott, MS

Matthew R. Jensen, MS

Aaron D. Norman, MPH

Kimberly A. Bertrand, ScD

V. Shane Pankratz, PhD

Kathleen R. Brandt, MD

Daniel W. Visscher, MD

John Shepherd, PhD

Rulla M. Tamimi, ScD

Celine M. Vachon, PhD

Karla Kerlikowske, MD

**Corresponding Author:**

Yiwey Shieh, MD

Division of General Internal Medicine, University of California, San Francisco

1545 Divisadero Street, Suite 0320

San Francisco, CA 94115

[Yiwey.Shieh@ucsf.edu](mailto:Yiwey.Shieh@ucsf.edu)

**Figure S1**


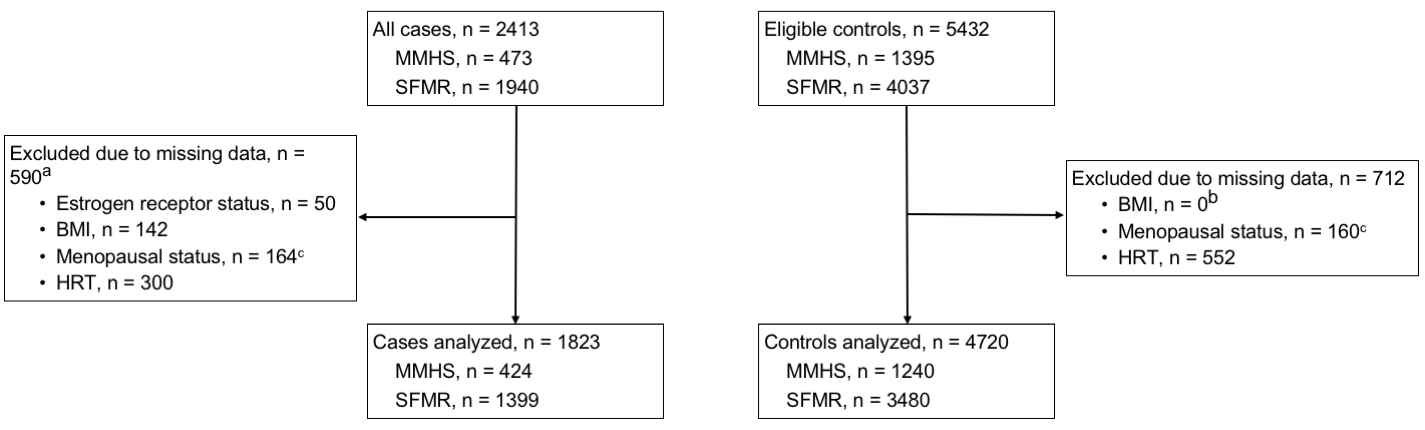


^a^ Some participants had missing values for more than one variable.

^b^ Controls were considered eligible if they were not missing BMI

^c^ Missing menopausal status was defined as: 1) hysterectomy without bilateral oophorectomy or surgical menopause and no concurrent HT use; or 2) menopause status unable to be determined based on available information.

**Table S1**

| **Characteristic** | **Mayo Cases^a^**  **(n = 424)** | **Mayo Controls^a^**  **(n = 1240)** | **UCSF Cases^b^**  **(n = 1399)** | **UCSF Controls^b^**  **(n = 3480)** |
| --- | --- | --- | --- | --- |
| Age at diagnosis, years (median, IQR) | 66 (57-74) |  | 58 (50-68) |  |
| Age at mammogram, years (median, IQR) | 60 (51-68) | 59 (51-68) | 55 (46-64) | 55 (46-64) |
| Race/Ethnicity, No. (%) |  |  |  |  |
| Caucasian | 422 (99.5) | 1229 (99.1) | 908 (64.9) | 2136 (61.4) |
| Asian | 0 (0.0) | 8 (0.6) | 301 (21.5) | 836 (24.0) |
| Hispanic/Latina | 2 (0.5) | 3 (0.2) | 67 (4.8) | 140 (4.0) |
| African-American | 0 (0.0) | 0 (0.0) | 69 (4.9) | 199 (5.7) |
| Multiracial | 0 (0.0) | 0 (0.0) | 34 (2.4) | 121 (3.5) |
| Other (not falling in categories above) | 0 (0.0) | 0 (0.0) | 20 (1.4) | 48 (1.4) |
| BMI, kg/m^2^ (median, IQR) | 27.7 (24.0-32.1) | 26.7 (23.6-31.1) | 24.1 (21.6-27.4) | 23.4 (21.3-26.6) |
| Menopausal status^a^ |  |  |  |  |
| Premenopausal | 99 (23.3%) | 290 (23.4%) | 511 (36.5%) | 1217 (35.0%) |
| Postmenopausal, estrogen use | 49 (11.6%) | 181 (14.6%) | 91 (6.5%) | 254 (7.3%) |
| Postmenopausal, estrogen + progestin use | 36 (8.5%) | 89 (7.2%) | 303 (21.7%) | 600 (17.2%) |
| Postmenopausal, no HT use | 240 (56.6%) | 680 (54.8%) | 494 (35.3%) | 1409 (40.5%) |
| Positive family history of breast cancer, No. (%) | 113 (26.7%) | 259 (20.9%) | 291 (20.8%) | 453 (13.0%) |
| Percent density (median, IQR) | 26.3 (15.3-40.5) | 22.2 (11.1-37.8) | 34.0 (17.7-50.9) | 25.8 (12.3-41.8) |
| Estrogen receptor status, No. (%) |  |  |  |  |
| Positive | 374 (88%) | NA | 1164 (83%) | NA |
| Negative | 50 (12%) | NA | 235 (17%) | NA |

Abbreviations: BMI, body mass index; H.T., hormone therapy, IQR, interquartile range (25^th^, 75^th^ percentile)

^a^ Controls and cases were matched on age, menopausal status at the time of index mammogram, year of examination, and state of residence

^b^ Controls and cases were matched on age, menopausal status at the time of index mammogram, year of examination, and screening facility

**Table S2**

|  | **Cases** | **Controls** | **Percent density OR per S.D. (95% CI)^a^** | **P_het_^b^** |
| --- | --- | --- | --- | --- |
| **Premenopausal** |  |  |  | 0.81 |
| ER-positive cancer | 505 | 1507 | 1.45 (1.29-1.64) |  |
| ER-negative cancer | 105 | 1507 | 1.52 (1.19-1.94) |  |
| **Postmenopausal HT user** |  |  |  | 0.41 |
| ER-positive cancer | 411 | 1124 | 1.54 (1.35-1.75) |  |
| ER-negative cancer | 68 | 1124 | 1.37 (1.05-1.79) |  |
| **Postmenopausal HT non-user** |  |  |  | 0.56 |
| ER-positive cancer | 622 | 2089 | 1.28 (1.16-1.41) |  |
| ER-negative cancer | 112 | 2089 | 1.20 (0.98-1.47) |  |

Abbreviations: CI, confidence interval; ER, estrogen receptor; HT, hormone therapy; OR, odds ratio; S.D., standard deviation

^a^ Odds ratios estimated from polytomous multivariable logistic regression model adjusted for age, study. Standard deviation of square root-transformed percent density = 2.0.

^b^ P-value of heterogeneity of BMI by subtype association

**Table S3**

|  | **Normal/underweight**  **BMI <25 kg/m^2^** | | | **Overweight**  **BMI 25-29.9 kg/m^2^** | | | **Obese**  **BMI ≥ 30 kg/m^2^** | | |
| --- | --- | --- | --- | --- | --- | --- | --- | --- | --- |
|  | **Cases** | **Controls** | **Percent density OR per S.D.**  **(95% CI)^a^** | **Cases** | **Controls** | **Percent density OR per S.D.**  **(95% CI)^a^** | **Cases** | **Controls** | **Percent density OR per S.D.**  **(95% CI)^a^** |
| **Premenopausal^b^** |  |  |  |  |  |  |  |  |  |
| ER-positive cancer | 325 | 979 | 1.90 (1.57-2.29) | 113 | 333 | 1.23 (0.95-1.59) | 67 | 195 | 1.69 (1.23-2.32) |
| ER-negative cancer | 58 | 979 | 1.51 (1.04-2.21) | 32 | 333 | 1.93 (1.19-3.13) | 15 | 195 | 3.04 (1.57-5.89) |
| *P_het_^e^* |  |  | 0.27 |  |  | 0.08 |  |  | 0.09 |
| **Postmenopausal HT user^c^** |  |  |  |  |  |  |  |  |  |
| ER-positive cancer | 240 | 678 | 1.71 (1.42-2.05) | 109 | 292 | 1.60 (1.22-2.09) | 62 | 154 | 1.83 (1.30-2.57) |
| ER-negative cancer | 41 | 678 | 1.34 (0.92-1.94) | 15 | 292 | 2.56 (1.27-5.19) | 12 | 154 | 1.41 (0.76-2.65) |
| *P_het_^e^* |  |  | 0.22 |  |  | 0.20 |  |  | 0.44 |
| **Postmenopausal** **HT non-user^d^** |  |  |  |  |  |  |  |  |  |
| ER-positive cancer | 228 | 1029 | 1.51 (1.27-1.80) | 219 | 648 | 1.53 (1.27-1.84) | 175 | 412 | 1.69 (1.37-2.10) |
| ER-negative cancer | 51 | 1029 | 1.23 (0.89-1.70) | 35 | 648 | 1.29 (0.86-1.93) | 26 | 412 | 1.76 (1.12-2.75) |
| *P_het_^e^* |  |  | 0.25 |  |  | 0.42 |  |  | 0.88 |

Abbreviations: BMI, body mass index; CI, confidence interval; ER, estrogen receptor; HT, hormone therapy; OR, odds ratio; S.D., standard deviation

^a^ Odds ratios estimated from polytomous multivariable logistic regression model adjusted for age, study, and continuous BMI within BMI strata. Standard deviation of square root-transformed percent density = 2.0.

^b^ P_int_ for BMI-percent density interaction = 0.03, assuming ordinal trend across BMI categories

^c^ P_int_ for BMI-percent density interaction = 0.47, assuming ordinal trend across BMI categories

^d^ P_int_ for BMI-percent density interaction = 0.77, assuming ordinal trend across BMI categories

^e^ P-value of heterogeneity of association between percent density and ER subtypes within BMI group

**Table S4**

|  | **Normal/underweight**  **BMI < 25 kg/m^2^** | | | | **Overweight/obese**  **BMI ≥ 25 kg/m^2^** | | | |
| --- | --- | --- | --- | --- | --- | --- | --- | --- |
|  | **Cases** | **Controls** | **Percent density**  **OR per S.D. (95% CI)^a^** | **P_het_^b^** | **Cases** | **Controls** | **Percent density**  **OR per S.D. (95% CI)^a^** | **P_het_^b^** |
| **Premenopausal^c^** |  |  |  | 0.18 |  |  |  | 0.02 |
| ER-positive cancer | 217 | 657 | 1.84 (1.46-2.32) |  | 115 | 368 | 1.32 (1.04-1.67) |  |
| ER-negative cancer | 36 | 657 | 1.32 (0.83-2.09) |  | 34 | 368 | 2.36 (1.50-3.71) |  |
| **Postmenopausal HT user^d^** |  |  |  | 0.07 |  |  |  | 0.77 |
| ER-positive cancer | 207 | 538 | 1.75 (1.43-2.15) |  | 144 | 344 | 1.69 (1.34-2.12) |  |
| ER-negative cancer | 29 | 538 | 1.16 (0.76-1.78) |  | 21 | 344 | 1.56 (0.94-2.60) |  |
| **Postmenopausal HT non-user^e^** |  |  |  | 0.40 |  |  |  | 0.46 |
| ER-positive cancer | 163 | 669 | 1.41 (1.15-1.71) |  | 296 | 789 | 1.50 (1.28-1.76) |  |
| ER-negative cancer | 31 | 669 | 1.17 (0.78-1.74) |  | 37 | 789 | 1.29 (0.88-1.90) |  |

Abbreviations: BMI, body mass index; CI, confidence interval; ER, estrogen receptor; HT, hormone therapy; OR, odds ratio; S.D., standard deviation

^a^ Odds ratios estimated from polytomous multivariable logistic regression models adjusted for age, study. Standard deviation of square root-transformed percent density = 2.0.

^b^ P-value of heterogeneity of association between percent density and ER subtypes within BMI groups

^c^ P_int_ = 0.02 for BMI-percent density interaction within premenopausal women.

^d^ P_int_ = 0.53 for BMI-percent density interaction within postmenopausal HT users.

^e^ P_int_ = 0.87 for BMI-percent density interaction within postmenopausal HT non-users.

**Table S5**

|  | **Normal/underweight**  **BMI < 25 kg/m^2^** | | | | **Overweight/obese**  **BMI ≥ 25 kg/m^2^** | | | |
| --- | --- | --- | --- | --- | --- | --- | --- | --- |
|  | **Cases** | **Controls** | **Percent density**  **OR per S.D. (95% CI)^a^** | **P_het_^b^** | **Cases** | **Controls** | **Percent density**  **OR per S.D. (95% CI)^a^** | **P_het_^b^** |
| **Premenopausal^c^** |  |  |  | 0.24 |  |  |  | 0.02 |
| ER-positive cancer | 325 | 979 | 1.93 (1.60-2.33) |  | 180 | 528 | 1.34 (1.11-1.62) |  |
| ER-negative cancer | 58 | 979 | 1.52 (1.04-2.22) |  | 47 | 528 | 2.12 (1.46-3.09) |  |
| **Postmenopausal HT user^d^** |  |  |  | 0.25 |  |  |  | 0.88 |
| ER-positive cancer | 240 | 678 | 1.72 (1.43-2.06) |  | 171 | 446 | 1.61 (1.31-1.97) |  |
| ER-negative cancer | 41 | 678 | 1.36 (0.94-1.98) |  | 27 | 446 | 1.66 (1.06-2.61) |  |
| **Postmenopausal HT non-user^e^** |  |  |  | 0.25 |  |  |  | 0.66 |
| ER-positive cancer | 228 | 1029 | 1.52 (1.28-1.80) |  | 394 | 1060 | 1.50 (1.31-1.72) |  |
| ER-negative cancer | 51 | 1029 | 1.24 (0.89-1.72) |  | 61 | 1060 | 1.40 (1.05-1.88) |  |

Abbreviations: BMI, body mass index; CI, confidence interval; ER, estrogen receptor; HT, hormone therapy; OR, odds ratio; S.D., standard deviation

^a^ Odds ratios estimated from polytomous multivariable logistic regression models adjusted for age, study, year of mammogram. Standard deviation of square root-transformed percent density = 2.0.

^b^ P-value of heterogeneity of association between percent density and ER subtypes within BMI groups

^c^ P_int_ = 0.007 for BMI-percent density interaction within premenopausal women.

^d^ P_int_ = 0.57 for BMI-percent density interaction within postmenopausal HT users.

^e^ P_int_ = 0.89 for BMI-percent density interaction within postmenopausal HT non-users.
